# Molecular features of a Huntington's disease knock-in minipig

**DOI:** 10.1242/dmm.052803

**Published:** 2026-05-26

**Authors:** Anastasiia Kolesnikova, Kirupa Sathasivam, Solaleh Khoramian Tusi, Christian Landles, Georgina F. Osborne, Marina Kovalenko, Tammy Gillis, Eva Kamenná, David Sekáč, Duong The Nguyen, Ivona Valeková, Štefan Juhás, Jana Juhásová, Božena Levinská, Monika Baxa, Jan Motlík, Jiří Klíma, David Howland, Vanessa C. Wheeler, Gillian P. Bates, Zdenka Ellederova

**Affiliations:** ^1^ Laboratory of Cell Regeneration and Plasticity, Institute of Animal Physiology and Genetics, Czech Academy of Sciences, Libechov 27721, Czech Republic; ^2^ Department of Cell Biology, Faculty of Science, Charles University in Prague, Prague 12800, Czech Republic; ^3^ Huntington's Disease Centre and Department of Neurodegenerative Disease, Queen Square Institute of Neurology, University College London, London WC1N 3BG, UK; ^4^ Molecular Neurogenetics Unit, Center for Genomic Medicine, Massachusetts General Hospital, Boston, MA 02114, USA; ^5^ Department of Neurology, Massachusetts Hospital and Harvard Medical School, Boston, MA 02114, USA; ^6^ CHDI Management, Inc., the company that manages the scientific activities of CHDI Foundation Inc., Princeton, NJ 08540, USA

**Keywords:** Huntington's disease, Knock-in minipig model, Somatic instability, *HTT* transcripts, HTT1a protein

## Abstract

Huntington's disease is caused by a CAG expansion in the *HTT* gene, leading to somatic repeat instability, alternative processing of *HTT* pre-mRNA, and mutant huntingtin protein production. To model these features, we generated a knock-in minipig (KI-85Q-HD) carrying a (CAG)82CAA(CAG)2 repeat in the endogenous *HTT* locus. To evaluate this, we quantified somatic expansion in various tissues using small pool- and bulk-PCR; detected *HTT1a*, an aberrantly spliced HTT transcript, using 3′ rapid amplification of cDNA ends and quantitative PCR; and assessed mutant huntingtin protein isoforms using homogeneous time-resolved fluorescence assays. Moderate levels of tissue-specific and age-dependent somatic expansion were observed, highest in the caudate nucleus, kidney and spleen, and detectable in blood cells. We confirmed the presence of *HTT1a* transcripts terminating at a cryptic polyadenylation site in *HTT* intron 1, and detected soluble full-length mutant HTT and HTT1a proteins across brain regions and peripheral tissues, while aggregated HTT1a was only detected in the cortex. These results indicate that KI-85Q-HD minipigs exhibit molecular features of Huntington's disease at a pre-symptomatic stage and may serve as a platform for assessing therapeutic distribution and potency.

## Teaser

**Summary:** Generation of a knock-in minipig model carrying an expanded *HTT* allele that recapitulates early molecular characteristics of Huntington's disease provides a valuable platform for evaluating emerging therapeutic approaches.

## INTRODUCTION

Huntington's disease (OMIM #143100) is a progressive and fatal neurodegenerative disorder caused by an expanded CAG repeat (≥36 units) in the first exon of the huntingtin (*HTT*) gene (The Huntington's Disease Collaborative Research Group, 1993), which is translated into an extended glutamine tract in the huntingtin (HTT) protein. Age at motor onset of Huntington's disease is inversely correlated with CAG repeat length [Duyao et al., 1993; Genetic Modifiers of Huntington's Disease (GeM-HD) Consortium, 2019; Langbehn et al., 2004]. It is characterized by progressive motor dysfunction that includes involuntary choreatic movements and impaired balance, as well as cognitive decline and psychiatric disturbances, and usually manifests in middle age (Bates et al., 2015; Martin and Gusella, 1986). The brain is the most severely affected organ, with medium-sized spiny neurons in the striatum and pyramidal neurons in the cortex particularly susceptible to degeneration (Ferrante et al., 1985; Vonsattel et al., 1985). In addition to neuronal loss, Huntington's disease is associated with progressive white matter alterations, including early microstructural changes detectable before clinical onset (Casella et al., 2020; Rosas et al., 2010).

A molecular hallmark of Huntington's disease is somatic CAG repeat instability, characterized by tissue/cell type-specific, expansion-biased repeat lengthening over time (Gonitel et al., 2008; Handsaker et al., 2025; Lee et al., 2010; Mätlik et al., 2024; Mouro Pinto et al., 2020; Pressl et al., 2024; Wheeler et al., 1999). Large expansions are detected in the striatum, while the liver represents the most unstable peripheral tissue in both mouse and human models. Larger somatic expansions in postmortem brain correlate with an earlier disease onset, and human genome-wide association studies, single-cell analyses and mouse functional studies directly support somatic expansion as a significant contributor to the timing of onset of clinical phenotypes [Genetic Modifiers of Huntington's Disease (GeM-HD) Consortium, 2019; Handsaker et al., 2025; Mouro Pinto et al., 2025; Swami et al., 2009].

Another molecular hallmark in Huntington's disease is the alternative processing of mutant *HTT* pre-mRNA, generating an exon 1-intron 1 transcript (*HTT1a*) that is translated to produce the highly pathogenic HTT exon 1 protein (HTT1a) (Neueder et al., 2017; Sathasivam et al., 2013). The amount of HTT1a generated increases with increasing CAG repeat length, whereas the level of full-length mutant HTT decreases (Landles et al., 2024). HTT1a is highly aggregation prone (Landles et al., 2010) and the presence of these aggregates correlates with disease progression in Huntington's disease mouse models (Yang et al., 2020). Recent preclinical data indicate that selective knockdown of *HTT1a* has a more beneficial impact on molecular phenotypes than knockdown of full-length *HTT* (Bragg et al., 2025 preprint; Papadopoulou et al., 2025 preprint).

Molecular studies of Huntington's disease have primarily been performed in mouse models and postmortem human brain tissue, including research on the generation of *HTT1a* and CAG repeat instability (Franich et al., 2019; Mouro Pinto et al., 2020, 2025; Neueder et al., 2017; Sathasivam et al., 2013). The development of gene therapy and other innovative Huntington's disease treatment strategies has necessitated the development of robust preclinical models to conduct therapeutic efficacy and safety studies. Physiologically and anatomically, pigs are very similar to humans, especially in terms of brain size and structure, which facilitated the analysis of detailed studies (Howland et al., 2020; Vodička et al., 2005).

A transgenic minipig model of Huntington's disease expressing the N-terminal 548-amino-acid part of human *HTT*, encoded by a stable 124 CAG/CAA repeat, demonstrates mild neuropathological features of Huntington's disease, including mutant HTT accumulation, white matter demyelination and microglial activation (Ardan et al., 2019; Askeland et al., 2018; Baxa et al., 2019). This transgenic minipig model proved useful in the preclinical study of huntingtin lowering. The study showed that AAV5-miHTT treatment enables widespread vector distribution, long-term expression, and significant reduction of human mutant HTT in the minipig brain (Evers et al., 2018; Vallès et al., 2021), supporting the clinical development of AMT-130 by uniQure (ClinicalTrials.gov identifier NCT04120493). A humanized knock-in (KI) Huntington's disease minipig model has recently been generated whereby the porcine exon 1 of *HTT* was replaced with human *HTT* exon 1 containing a pure repeat of 150 CAGs, and these minipigs produce the *HTT1a* transcript (Tong et al., 2023). They show intergenerational CAG repeat instability (Yan et al., 2018) but little evidence of substantial somatic instability (Bai et al., 2021). This model has been used to test a therapeutic approach of Cas9-mediated replacement of the expanded CAG repeat (Yan et al., 2023).

Here, we present a study describing the detailed characterization of a new KI minipig model, KI-85Q-HD, which was developed with a CAG expansion encoding ∼85 glutamines in the endogenous porcine *HTT* locus. We have analyzed the key molecular pathogenic mechanisms of somatic CAG repeat instability and assessed the production of the HTT1a protein that will guide the use of this model in preclinical therapeutic testing.

## RESULTS

### Validation and characterization of KI-85Q-HD minipigs

**Fig. 1.**
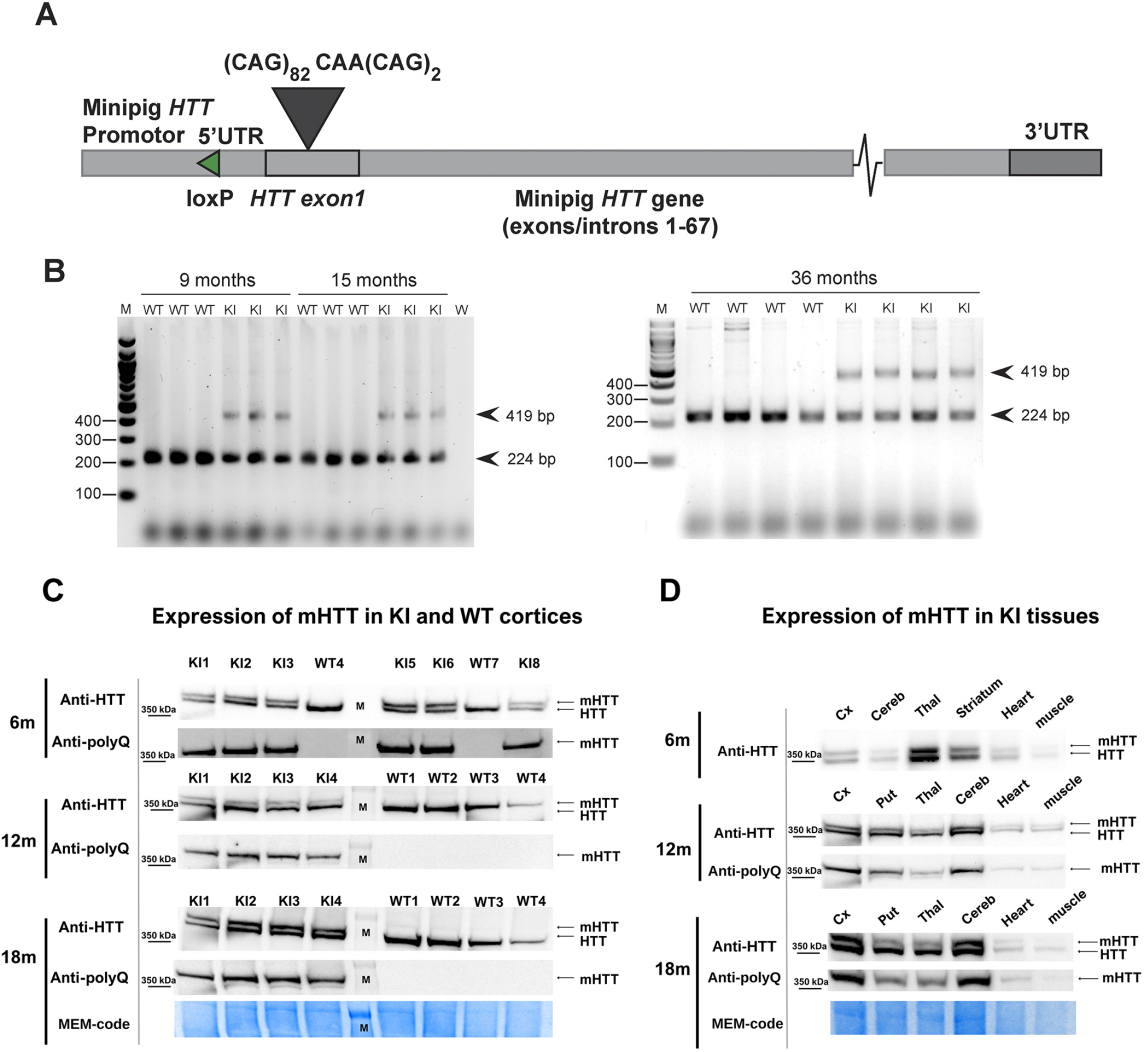
**Characterization of the KI-85Q-HD minipig model.** (A) Schematic of the KI mutation in the minipig *HTT* gene with the insertion of the expanded CAG repeat: (CAG)_82_CAA(CAG)_2_. (B) PCR of striatal cDNA or genomic DNA from the F0 and F2 generation of minipigs at 9, 15 and 36 months of age confirms the genotypes. Genotyping primers flank the CAG repeat-containing region in *HTT* exon 1. The molecular-mass marker (Hi-marker; M) is a 100 bp DNA ladder. W, water control. (C) Detection of the HTT protein (antibody EPR5526) and mutant HTT (polyQ antibody 1C2 or 3B5H10) in the cortex of WT and KI minipigs at 6, 12 and 18 months of age. MemCode staining was used as a loading control. Full western blot membranes and MemCode-stained loading controls are shown in Fig. S1A-F. (D) Detection of the HTT protein (antibody EPR5526) and mutant HTT (mHTT; polyQ antibody 1C2 or 3B5H10) in cortex (Cx), putamen (Put), thalamus (Thal), cerebellum (Cereb), heart, muscle and striatum of KI minipigs at 6, 12 and 18 months of age. MemCode staining was used as a loading control. Full western blot membranes and MemCode-stained loading controls are shown in Fig. S2A-C. m, months.

A new KI minipig model (KI-85Q-HD) was generated by targeted insertion of an expanded CAG repeat that encodes an 85Q repeat into exon 1 of the endogenous *HTT* gene under its native promoter (Fig. 1A), enabling physiological expression of mutant HTT. To validate the model, we first confirmed the presence and expression of the expanded CAG repeat. Genotyping by PCR and gel electrophoresis of cDNA samples from the striatum of F0 (9 and 15 months of age) and F2 (36 months of age) generations revealed a PCR product of 224 bp, corresponding to the 14-CAG repeat-containing normal allele, and a PCR product of 419 bp, corresponding to the 82 pure CAG repeats in the expanded allele (Fig. 1B). These results validated the successful integration and expression in the RNA of the expanded CAG repeat. We then investigated the expression of full-length HTT proteins levels in cortical samples from KI-85Q-HD minipigs at 6, 12 and 18 months of age by western blotting (Fig. 1C). These time points represent early stages of postnatal maturation in the minipig and were chosen to validate the early stable expression of mHTT as a molecular baseline for the young adult KI-85Q-HD model. Total HTT and mutant HTT were detected with the EPR5526 antibody, while mutant HTT was specifically detected using polyglutamine-binding antibodies 1C2 or 3B5H10. Mutant HTT expression was consistently observed across all ages, without significant temporal variation. These results confirm stable production of the mutant protein, supporting the reliability of the KI-85Q-HD minipig model for longitudinal studies of Huntington's disease pathogenesis. Next, we investigated the tissue-specific distribution of mutant HTT expression in KI-85Q-HD minipigs at 6, 12 and 18 months (Fig. 1D). Western blot analysis showed that mutant HTT and wild-type (WT) HTT were expressed most highly in brain regions. In contrast, peripheral tissues, such as the heart and muscle, exhibited lower levels of mutant HTT expression. There was no obvious change in the pattern of regional expression over time. The relative levels of mutant HTT seem to parallel those of WT HTT across these tissues, as expected, since both are driven by the endogenous promoter.

As shown in Fig. 1, the size of the expanded alleles in the cDNA did not obviously differ between animals on agarose gels, suggesting that the repeat might be relatively stable across generations. However, accurate repeat sizing revealed CAG lengths from 82 to 90 (Table S1), indicating some level of intergenerational repeat instability.

### Somatic instability of CAG repeats in KI-85Q-HD minipigs

**Fig. 2.**
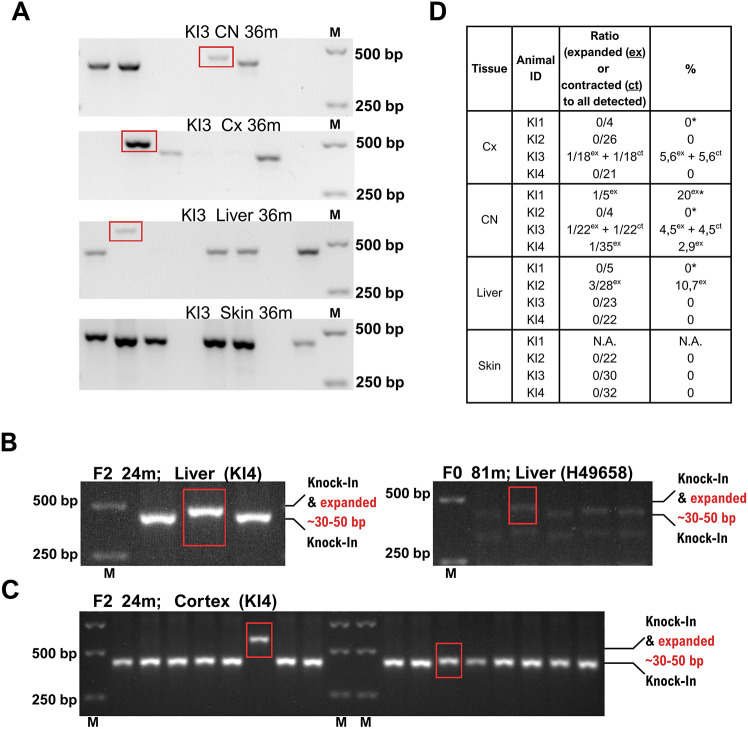
**Small pool-PCR analyses of somatic CAG repeat instability.** (A) KI allele-specific SP-PCR of genomic DNA from the cortex (Cx), caudate nucleus (CN), liver and skin from pig ID KI3 at 36 months of age. The expanded PCR products are marked with a red box. (B) Re-analysis of KI allele-specific SP-PCR products from genomic DNA from the liver of a 24-month-old pig (KI4) (left) and KI allele-nonspecific SP-PCR products from genomic DNA from the liver of an 81-month-old pig (H49658) (right). (C) Re-analysis of KI allele-specific SP-PCR products from genomic DNA from the cortex of KI4 at 24 months of age. Allele products were amplified from 30 genome equivalents or fewer, and expanded alleles are marked by red boxes. Minor variations in PCR product length (e.g. noted by red rectangle) are frequent. They are evaluated by assessing whether they are expanded or contracted relative to most of the alleles. (D) The expanded allele frequencies as a ratio of expanded/contracted alleles to all amplified KI alleles. Asterisks indicate samples in which only a small number of KI alleles could be evaluated. CN, caudate nucleus; ct, contracted alleles; Cx, cortex; ex, expanded alleles; m, months; M, 1 kb DNA marker; N.A., not applicable.

To investigate somatic instability in the KI-85Q-HD minipigs, we developed sensitive KI allele-specific and nonspecific small-pool PCR (SP-PCR) assays that would allow us to detect relatively rare somatic repeat length changes, consisting of two sequential (nested) PCR reactions to detect expanded KI CAG repeats in genomic DNA from as few as 30 genome equivalents. KI allele-specific SP-PCR was performed on genomic DNA isolated from the liver, skin, caudate nucleus and cortex of a 36-month-old KI-85Q-HD minipig. Expanded KI alleles were detected in liver, caudate nucleus and cortex, whereas no expansions were observed in skin (Fig. 2A). To further investigate somatic CAG repeat instability in another tissue, SP-PCR was performed on liver genomic DNA from a 24-month-old (KI4, F2) and an 81-month-old (H49658, F0 generation) KI-85Q-HD minipig (Fig. 2B). Some KI products document somatic expansion of approximately 30-50 bp difference, as indicated by a red rectangle in both F0 and F2 generation animals. To further evaluate somatic instability in the cortex, KI allele-specific SP-PCR was performed on genomic DNA isolated from the frontal cortex of the KI4 (24 months old, F2) minipig (Fig. 2C). The KI allele (∼500 bp) was amplified across most reactions, and a distinct expanded allele, indicated by a red box, was detected in one reaction. Although minor variations in PCR product size among the amplified alleles were observed, their sizes could not be accurately determined due to the resolution limits of standard agarose gel electrophoresis. To quantify somatic CAG repeat instability, we therefore visually assessed whether the PCR products were expanded or contracted relative to most KI alleles and calculated the ratio of expanded and contracted alleles relative to all amplified KI alleles in cortex, caudate nucleus, liver and skin of four 36-month-old KI-85Q-HD minipigs (Fig. 2D). In the cortex, somatic instability was detected only in one animal (KI3), with 5.6% of sampled alleles expanded and 5.6% contracted. In the caudate nucleus, comparable frequencies (4.5% of sampled alleles) of expanded and contracted alleles were observed in animal KI3, while we detected a single expansion event in two other animals KI1 (20% sampled alleles) and KI4 (2.9% of sampled alleles). In the liver, three expansion events (10.7% of sampled alleles) were detected in only one animal (KI2). No instability was detected in the skin across all animals analyzed. Although the limited and variable allele sampling between animals and tissues precludes our ability to draw strong conclusions, overall the data suggest that there are low levels of somatic instability in the KI-85Q-HD minipigs but indicate that the instability may exhibit tissue specificity, e.g. the caudate nucleus shows expanded alleles in three of the four animals tested, while no instability was detected in the skin of any animal.

**Fig. 3.**
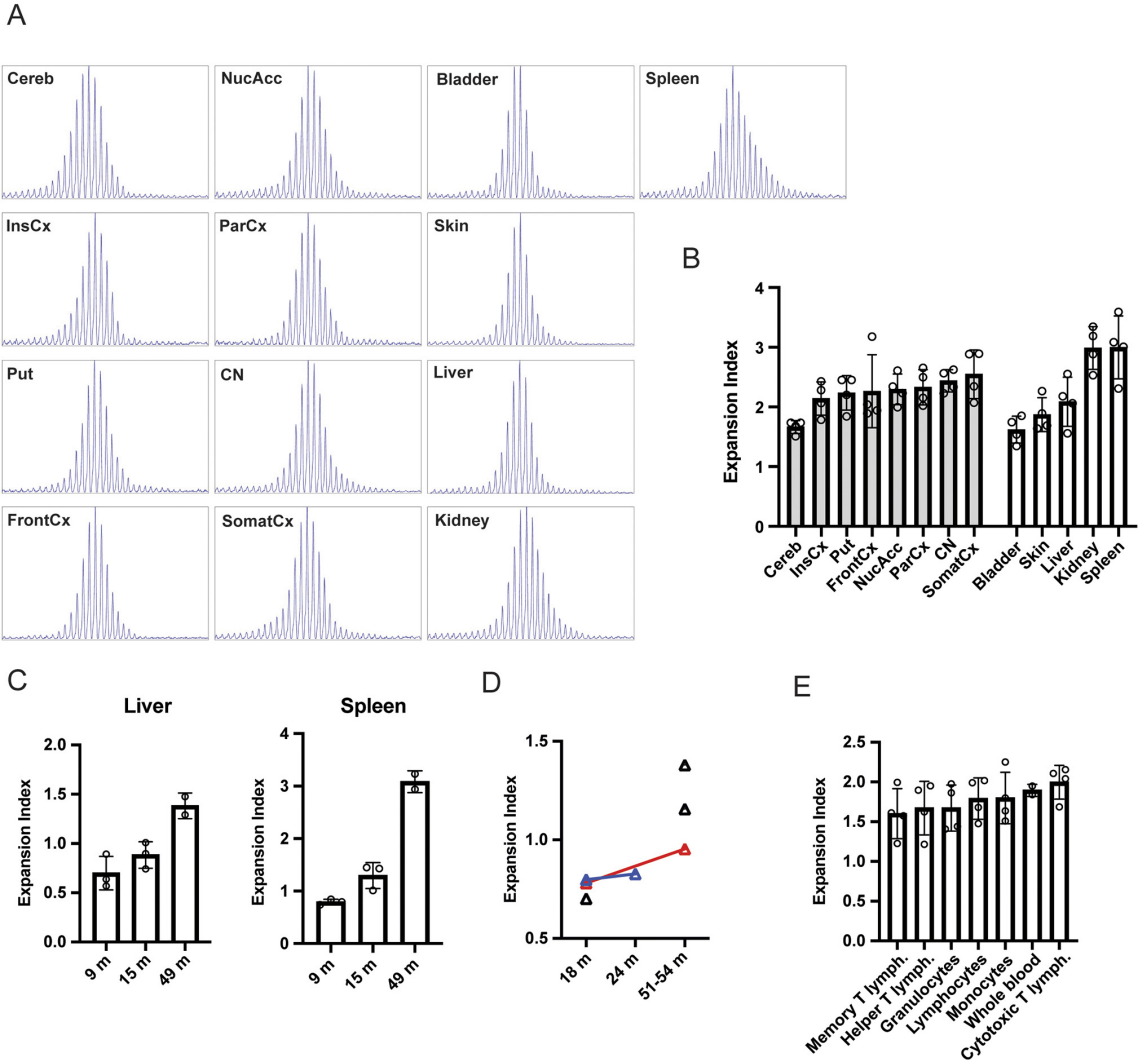
**Fragment analysis of somatic CAG repeat instability in KI-85Q-HD minipigs.** (A) GeneMapper profiles of various brain regions and peripheral tissues from a 36-month-old minipig. (B) Expansion indices derived from the fragment analysis of genomic DNA reveal tissue-specific differences in CAG repeat expansion across brain regions (cerebellum, insular cortex, putamen, frontal cortex, parietal cortex, somatosensory cortex, caudate nucleus, nucleus accumbens) and peripheral tissues (bladder, skin, liver, kidney, spleen). One-way ANOVA: *P*<0.0001; Dunnett's multiple comparisons test, relative to cerebellum: caudate nucleus *P*=0.03, somatosensory cortex *P*=0.0096, kidney *P*<0.0001, spleen *P*<0.0001. Data represent mean±s.d.; *n*=4 (animals KI-KI4; Table S1). CAG lengths (cerebellum) range from 83 to 88. (C) Expansion indices measured in liver and spleen from animals aged 9 months (*n*=3; CAG 81-83), 15 months (*n*=3; CAG 83-85) and 49 months (*n*=2; CAG 82) show age-dependent increases in somatic instability (one-way ANOVA: liver *P*=0.01, spleen *P*=0.001). Data shown are mean±s.d. (D) Expansion indices in semen DNA from five animals (CAG 82-85) aged 18-54 months indicate a trend towards age-related expansion (one-way ANOVA: *P*=0.07). Red and blue symbols indicate longitudinally sampled animals; black symbols represent single time-point samples. (E) Expansion indices in sorted blood cell populations and whole blood from 27-month-old minipigs (*n*=4; CAG 81-87). Memory T lymphocytes are CD4^+^CD8^+^, helper T lymphocytes are CD4^+^ and cytotoxic T lymphocytes are CD8^+^. Data shown are mean±s.d. Cereb, cerebellum; CN, caudate nucleus; FrontCx, frontal cortex; InsCx, insular cortex; lymph., lymphocytes; m, month; NucAcc, nucleus accumbens; ParCx, parietal cortex; Put, putamen; SomatCx, somatosensory cortex.

To test further the extent and tissue specificity of CAG repeat instability, we performed fragment sizing analyses of fluorescently labeled CAG-containing PCR products analyzed by capillary electrophoresis amplified from ‘bulk’ genomic DNA in a wide range of brain regions, peripheral tissues, and cell types. We first analyzed instability in the 36-month-old KI animals in which we had performed SP-PCR (KI1, KI2, KI3, KI4). Examples of electropherograms show a broad distribution of peaks from the expanded allele, whereas the WT allele exhibited a very narrow repeat length distribution (Fig. 3A, Fig. S3), confirming the presence of somatically expanded KI alleles (PCR slippage largely generates the contraction peaks). Overall, and as suggested by the SP-PCR results, the degree of somatic expansion was moderate. Nevertheless, quantification of expansion indices revealed tissue-specific differences in somatic expansion (Fig. 3B). Amongst the brain regions, the cerebellum was more stable than the cortical regions (insular, frontal, parietal and somatosensory cortices), or the basal ganglia (putamen, nucleus accumbens, caudate nucleus). Amongst the peripheral tissues, the bladder, skin and liver were relatively stable compared to the kidney and spleen. In post-hoc multiple comparison tests relative to the cerebellum, significantly greater expansion was seen in caudate nucleus, somatosensory cortex, kidney and spleen. The tissue specificity was consistent with that observed by SP-PCR (nucleus accumbens≈frontal cortex>liver>skin). To evaluate age-related changes, expansion indices were measured in liver and spleen samples from minipigs at 9 months, 15 months and 49 months of age (Fig. 3C). In both tissues, somatic CAG expansion increased with age. As the variation in repeat length between animals indicated some degree of intergenerational repeat instability, we also quantified repeat instability in genomic DNA isolated from semen samples of five animals at 18 months, 24 months and 51-54 months (Fig. 3D). We collected longitudinal samples in two of the animals, one at 18 months and 51 months (red symbols) and one at 18 and 24 months (blue symbols). The remaining three animals had a single sample collected, either at 18, 51 or 54 months (black symbols). Across all the animals, the mean expansion index in semen trended towards an increase with age. In the two animals with repeat sampling, the expansion index increased longitudinally with age (from 0.798 at 18 months to 0.827 at 24 months, and from 0.780 at 18 months to 0.954 at 54 months). Finally, given the potential of peripheral blood as a biomarker in preclinical trials, we analyzed sorted blood cell types from four 27-month-old animals and whole blood in two of these animals (Fig. 3E). Expansion was measurable in blood, and we detected only very subtle differences between blood cell populations.

### Detection of the *HTT1a* transcript in the KI-85Q-HD minipig brain

**Fig. 4.**
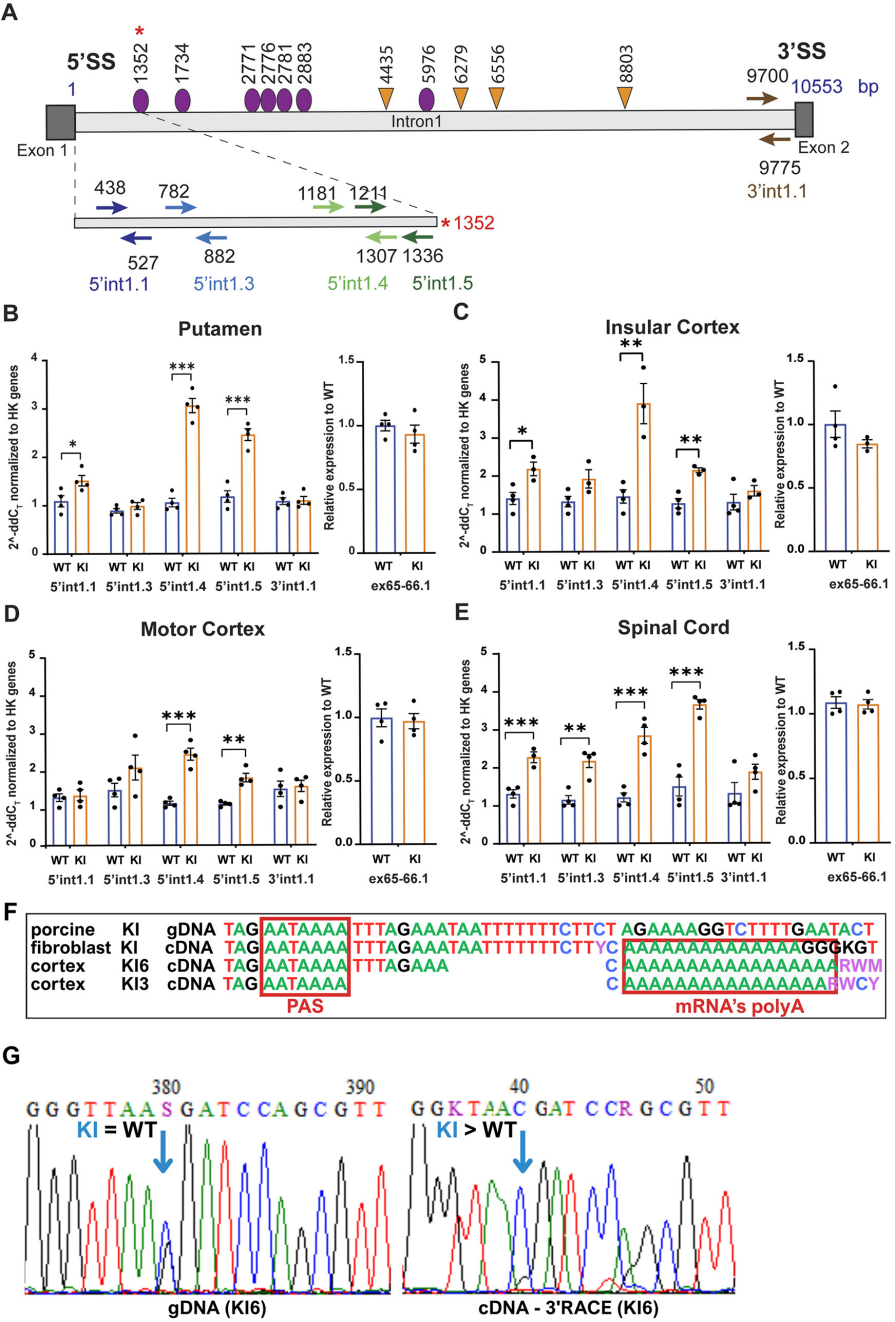
**Detection of the alternative processing of the *HTT* pre-mRNA by qPCR amplification and 3′RACE.** (A) Location of potential cryptic polyA sites in intron 1 of the minipig *HTT* gene. PCR primer sets were designed to amplify 5′ intron 1 sequences before the first polyA site and at the 3′ end of intron 1; the location of the PCR assays is given in base pairs from the 5′ end of intron 1. Ovals, AATAAA; triangles, ATTAAA. (B-E) qPCR signals within intron 1 compared to pre-mRNA levels in WT minipigs at 36 months of age for putamen (B), insular cortex (C), motor cortex (D) and spinal cord (E). The 5′int1.1, 5′int1.4 and 5′int1.5 assays indicated that transcript levels containing 5′ intron 1 sequences were higher than pre-mRNA levels in the WT minipigs, whereas the 3′int1.1 assay indicated that transcripts containing the 3′ end of intron 1 were at WT pre-mRNA levels. The level of full-length *HTT* was comparable between WT and KI-85-HD minipigs (ex65-66.1). *n*=4 per genotype. Statistical analysis was unpaired, two-tailed Student's *t*-test or one-way ANOVA with Tukey's post-hoc correction. Error bars are mean±s.e.m. **P<0.01, ***P<0.001. (F) 3′RACE products amplified from the first cryptic polyA site from the cortex and fibroblasts of a KI-85-HD minipig. (G) Sequencing of genomic DNA and 3′RACE products from cDNA from KI-85-HD minipig (KI6) cortex showing the presence of the SNP in phase with the mutant allele in the 3′RACE sample. Blue arrows indicate the SNP position (KI=WT, equal allele contribution in gDNA; KI>WT, predominant KI signal in 3′RACE cDNA). gDNA, genomic DNA; HK, housekeeping; PAS, polyadenylation site; mRNA′s polyA, polyA tail of mRNA; SS, splice site.

To determine whether the expanded CAG repeat in the KI-85Q-HD minipigs had led to the activation of cryptic polyadenylation (polyA) sites within *HTT* intron 1, we used the Softberry polyah and dnafsminer algorithms to map the potential polyA sites (Fig. 4A). There were 11 possible sites, seven of which had the consensus sequence AATAAA, and four had the sequence ATTAAA. Activation of any of these sites would lead to the production of *HTT1a* and an increase in mRNAs containing 5′ intron 1 sequences. Therefore, we designed a set of quantitative PCR (qPCR) assays located before the first polyA site, including an assay for the 3′ end of intron 1 for pre-mRNA levels and an assay for exons 65-66 to detect full-length *HTT* (Fig. 4A). qPCR was performed on cDNA or genomic DNA from the putamen, insular cortex, motor cortex and spinal cord from 36-month-old WT and KI-85Q-HD minipigs (Fig. 4B-E). An increase in 5′ intron 1 sequences, above pre-mRNA levels, was identified in all four brain regions, but interestingly, the greatest increase was seen with the qPCR assays that were closest to the first cryptic polyA sequence. This phenomenon was also observed for qPCR assays detecting *HTT1a* in human postmortem brain (Neueder et al., 2017). The 3′ intron 1 assay detected comparable levels of pre-mRNA between WT and KI-85Q-HD minipigs, and the level of the full-length *HTT* transcript was comparable between WT and KI-85Q-HD minipigs in all four brain regions (Fig. 4B-E). These results demonstrate that *HTT1a* transcript is generated in the KI-85Q-HD minipigs in all brain regions tested.

To determine whether the first cryptic polyA site was activated, we amplified 3′ rapid amplification of cDNA ends (3′RACE) cDNA derived from cortical RNA of the 36-month-old KI-85-HD minipigs (Fig. 4F). The 3′RACE product was approximately 600 bp in size, consistent with a truncated transcript terminating within intron 1. A reverse transcriptase negative control showed no amplification, confirming the absence of genomic DNA and non-specific products. To determine whether premature transcription termination occurred at the first polyA site, we analyzed 3′RACE cDNA sequences from cortex and fibroblast samples of KI-85Q-HD minipigs. As shown in Fig. 4F, analysis of cDNA sequences confirmed the presence of a truncated exon 1–intron 1 *HTT* transcript terminating within intron 1 with a polyA site (red box) followed by a polyadenylated tail (green box). To confirm that the truncated *HTT* transcript was derived from the mutated KI allele, we checked for a single nucleotide polymorphism (SNP) in genomic DNA and 3′RACE cDNA from the cortex of a heterozygous KI-85Q-HD minipig (Fig. 4G). Sequencing of genomic DNA revealed overlapping peaks corresponding to both WT and KI alleles at the SNP site. Sequencing of the 3′RACE product showed SNP base pattern associated with the mutant allele, demonstrating that cryptic polyA site activation had occurred on the KI allele.

### HTRF-based detection of soluble and aggregated HTT1a in CNS and peripheral tissues of KI-85Q-HD minipigs

**Fig. 5.**
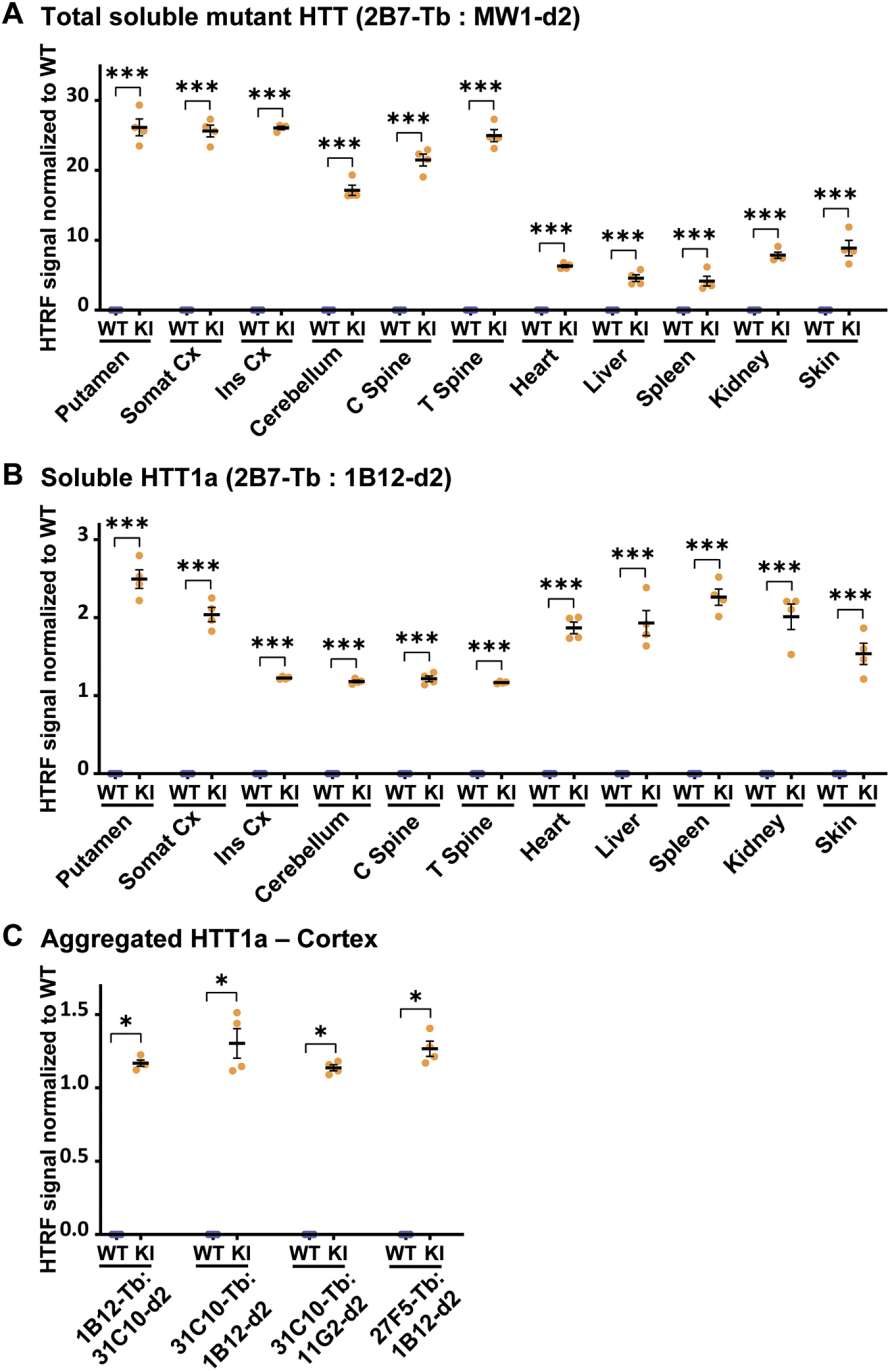
**The HTT1a protein is generated throughout the brain and peripheral tissues.** (A) HTRF analysis for mutant HTT (mutant full-length HTT and HTT1a) demonstrates that the level of mutant HTT was greater in CNS tissues than in the periphery. (B) HTRF analysis of the HTT1a protein shows that, within the brain, levels are highest in the putamen. HTT1a levels were higher in the periphery than in many of the CNS regions. (C) Aggregated HTT1a was only detected in the cortex. Aggregated HTT1a in the cortex could be detected with four independent assays. *n*=4 per genotype. Statistical analysis was one-way ANOVA with Bonferroni post-hoc correction. Error bars are mean±s.e.m. *P≤0.05, ***P≤0.001. C Spine, cervical spinal cord; Ins Cx, insular cortex; Somat Cx, somatosensory cortex; T Spine, thoracic spinal cord.

We applied homogeneous time-resolved fluorescence (HTRF) assays to compare the levels of HTT proteins between CNS regions and peripheral tissues. The 2B7 antibody binds to the first 17 amino acids of HTT, and MW1 is specific to an expanded polyglutamine repeat. Therefore, the 2B7-MW1 HTRF assay detects all mutant HTT proteins: full-length HTT, N-terminal proteolytic HTT fragment, and HTT1a. The 2B7-MW1 assay showed that the level of mutant HTT was higher in the CNS tissues than in the periphery, and the absence of a signal in the WT minipigs confirmed the specificity of the assay (Fig. 5A). 1B12 and 11G2 are neo-epitope antibodies for the C terminus of the HTT1a protein (Sapp et al., 2025 preprint). As with MW8, when combined with 2B7, 1B12 provides an HTRF assay (2B7-1B12) that is specific to HTT1a (Landles et al., 2021, 2024), but is more sensitive than 2B7-MW8. The level of HTT1a was found to be higher in the putamen and somatosensory cortex than in other CNS regions, but comparatively higher levels were also detected in peripheral tissues (Fig. 5B). HTRF assays can also be used to detect aggregated HTT. For human HTT, the 4C9 antibody to the proline-rich domain, combined with MW8, provides an assay that was specific for aggregated HTT1a (Smith et al., 2023). We found that the 4C9 antibody did not detect minipig HTT. Instead, we utilized two antibodies that detect epitopes close to the C terminus of HTT1a (31C10 and 27F5). Four available combinations of these two antibodies with the HTT1a-specific antibodies were tested (1B12-31C10, 31C10-1B12, 31C10-11G2, 27F5-1B12) and found to detect a signal above background in the cortex but not in any other brain region (Fig. 5C). The absence of an HTT aggregation signal may reflect the early disease state at 36 months, with the initiation of aggregation to detectable levels only having occurred in the cortex.

## DISCUSSION

**Fig. 6.**
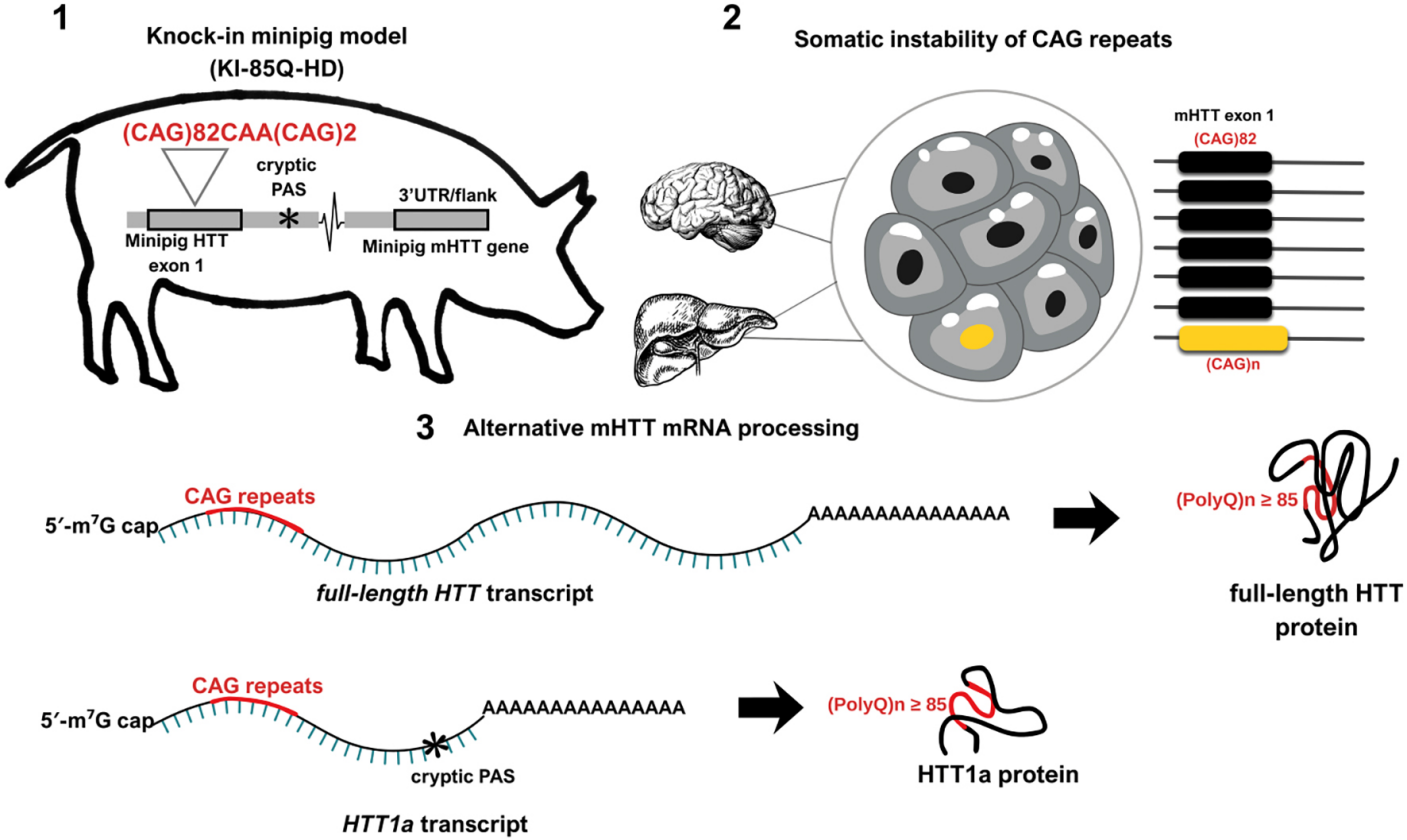
**Schematic overview of the molecular features modeled in the KI-85Q-HD minipig.** The KI-85Q-HD minipig recapitulates key molecular hallmarks of Huntington's disease: somatic CAG repeat instability and alternative processing of HTT pre-mRNA. (1) A (CAG)_82_CAA(CAG)_2_ repeat was introduced into the endogenous porcine *HTT* locus by CRISPR/Cas9 editing of exon 1. (2) The expanded allele undergoes tissue-specific and age-dependent somatic CAG repeat expansion, with the largest changes detected in the caudate nucleus, kidney and spleen. (3) Full-length *HTT* mRNA is translated into full-length HTT protein, while activation of a cryptic polyadenylation site (asterisk) within intron 1 generates the *HTT1a* transcript, which encodes the short, aggregation-prone HTT1a protein. CAG, cytosine, adenine, guanine; *HTT*, huntingtin gene; *HTT1a*, truncated HTT mRNA transcript generated by the alternative processing of intron 1; HTT1a, truncated N-terminal huntingtin protein translated from the *HTT1a* transcript; mHTT, mutant HTT; PAS, cryptic polyadenylation site; PolyQ, polyglutamine.

In this study, we present a novel KI-85Q-HD minipig model that endogenously expresses mutant *HTT* containing an expanded CAG tract and recapitulates key molecular hallmarks of Huntington's disease (Fig. 6). We confirmed correct targeted CAG repeat integration and the presence of full-length mutant HTT (∼350 kDa) in different tissues at levels comparable to WT HTT. In the KI-85Q-HD minipig model, mHTT expression was lower in peripheral tissues compared to the brain. This distribution aligns with other huntingtin patterns described in humans, and in mouse and rodent models (Li et al., 1993; Mangiarini et al., 1996) (Fig. 1). The abundance of the mutant HTT in specific brain regions was similar (e.g. cortex>putamen) to that observed in humans (Marques Sousa and Humbert, 2013). However, in peripheral regions, we detected a higher expression in the spleen, which diverges from mice and humans (HTT protein expression summary, The Human Protein Atlas, accessed 7 November 2025) suggesting possible species-specific differences. Overall, the KI-85Q-HD minipig expresses full-length mutant HTT at physiologically relevant levels and distributions, validating it as a genetically and biologically relevant large-animal model of Huntington's disease.

A hallmark of Huntington's disease is the tendency for the HTT CAG repeat to expand in somatic cells over time. The absolute somatic expansion observed in the KI-85Q-HD minipigs (at age 36-48 months) was low relative to levels in different lines of Huntington's disease KI mice with repeat lengths varying from ∼70 to 90 CAGs (collectively, ages spanning 9-27 months) (Kennedy et al., 2003; Shelbourne et al., 2007; Wheeler et al., 1999). Brain from a Huntington's disease individual with ∼87 CAGs exhibited even more extensive somatic expansion (Kennedy et al., 2003), although this would reflect expansion events accumulating over a longer period of time. Our findings correspond to observations in another Huntington's disease KI minipig model harboring ∼150 CAGs that exhibited low levels of somatic expansion at 4 months of age (Bai et al., 2021). Although the overall levels of expansion were low, we were able to quantify age-dependent effects and differences between tissues.

Brains of 36-month-old KI-85Q-HD animals showed modest somatic expansions and very subtle differences among the cortex, caudate, putamen and nucleus accumbens. In humans, extensive somatic expansion is apparent in the striatum and cortex, attributable to specific neuronal subtypes that can accumulate repeat lengths reaching into the hundreds (Handsaker et al., 2025; Lee et al., 2011; Mouro Pinto et al., 2020; Shelbourne et al., 2007; Wheeler et al., 2000). Huntington's disease mice also exhibit very high levels of expansion in the striatum (Lee et al., 2010). Negligible cerebellar expansions in the KI-85Q-HD minipig resemble those observed in humans and mice (Handsaker et al., 2025; Lee et al., 2011; Mouro Pinto et al., 2020; Wheeler et al., 2000).

In KI-85Q-HD minipig peripheral tissues, the spleen and kidney exhibited the most somatic expansion, exceeding levels seen in brain tissues, while the liver, skin and bladder had more moderate expansion. These tissue-specific differences, only in part, reflect those seen in humans and in mice as the liver is highly unstable both in humans and mice. The spleen is relatively stable in humans and very stable in mice (Handsaker et al., 2025; Lee et al., 2011; Mouro Pinto et al., 2020; Wheeler et al., 2000). The high instability in the spleen may reflect species-specific elevated expression of HTT in WT and KI minipig spleen and different porcine spleen anatomy or physiology compared to that of humans and rodents (Haley, 2017; Kirkland et al., 2022; Skydsgaard et al., 2021).

In humans, the CAG repeat in blood is relatively stable and increases modestly with age (Ciosi et al., 2019; Kacher et al., 2021). Similarly, a low level of somatic expansion was detected in whole blood and individual blood cell types in KI-85Q-HD minipigs. The reasons underlying the relatively modest somatic expansion in the KI-85Q-HD minipigs would be of interest to understand.

In addition to repeat expansion, another key molecular feature we examined was the alternative processing of *HTT* pre-mRNA to generate *HTT1a.* Using 3′RACE and quantitative RT-PCR, we detected polyadenylated *HTT* mRNA species terminating within intron 1, confirming the presence of the *HTT1a* transcript in our KI minipigs (Fig. 4). This finding aligns with evidence from rodent models and human Huntington's disease tissues that expanded CAG repeats can trigger the activation of cryptic polyA sites within intron 1 to generate the single-exon *HTT1a* transcript (Neueder et al., 2017; Sathasivam et al., 2013). *HTT1a* has been identified in all KI mouse models, as well as those transgenic for mutant full-length human *HTT*, where its presence correlates with CAG repeat length (Fienko et al., 2022; Landles et al., 2024). Our results provide evidence that the alternative processing of *HTT* pre-mRNA also occurs in a large-animal model of Huntington's disease. Similar to the humanized KI pig model (Tong et al., 2023), KI minipigs with pure 82 CAGs exhibited clear evidence of the production of *HTT1a* in minipig brain (and peripheral tissues), confirming that the model with the mutation leads to the same RNA processing event seen in humans (Fienko et al., 2022; Neueder et al., 2017). The production of *HTT1a* has significant pathogenic effects as it encodes the HTT1a protein that is highly prone to misfolding and aggregation. At 24 and 36 months of age, KI-85Q-HD minipigs did not display overt neurodegeneration, suggesting that the animals are still at an early, pre-symptomatic stage at the ages analyzed. Our minipig model may provide a platform for studying the effects of HTT1a in an organism that is closer to humans in size and lifespan. Moreover, confirming the presence of *HTT1a* in the minipig provides a basis for testing splicing-targeted therapeutic strategies, such as preventing intron 1 retention or blocking cryptic intronic polyadenylation (Bragg et al., 2025 preprint; Papadopoulou et al., 2025 preprint). The findings are consistent with HTT1a production being a mutation-driven feature of Huntington's disease, making our large-animal model a relevant and valuable tool for evaluating its regulation and potential therapeutic modulation.

Crucially, the *HTT1a* transcripts in the KI-85Q-HD minipig are translated into the toxic HTT1a protein. Using sensitive HTRF assays, we quantified both soluble and aggregated HTT1a in pig tissues. By 3 years of age, KI-85Q-HD minipigs showed measurable levels of soluble HTT1a in multiple brain regions (striatum, cortex, cerebellum), as well as in peripheral organs (liver, spleen) (Fig. 5A,B). The detection of aggregated HTT species in the minipigs was more problematic, as the 4C9 antibody that has been used in the human and KI mouse studies did not detect an epitope in the minipigs. Instead, we established an assay between antibodies close to the C terminus of HTT1a and 1B12 (an HTT1a neo-epitope antibody). Unfortunately, all of these assays were very weak and, although we did consistently detect aggregated HTT in the cortex at 3 years of age (Fig. 5C), more sensitive assays may have identified evidence of HTT aggregation in other brain regions. These findings demonstrate that the truncated HTT1a protein is produced and that at 3 years of age, the aggregation of HTT1a has been initiated. The detection of aggregated HTT1a primarily in the cortex is consistent with established human Huntington's disease pathology. As reported by Gutekunst et al. (1999), huntingtin aggregates, particularly neuropil aggregates, are significantly more abundant in the cerebral cortex than in the striatum, even in pre-symptomatic individuals. Interestingly, although the striatum is the most severely affected region in terms of neuronal loss, it often shows surprisingly low densities of visible aggregates compared to cortical layers. Our findings in the KI-85Q-HD minipig model align with this spatial gradient, suggesting that cortical HTT1a accumulation is an early molecular hallmark that can precede or occur independently of regional neurodegeneration. The analysis of older animals with more-sensitive assays may lead to the detection of HTT aggregation in other brain regions. The situation is similar in Huntington's disease KI mice. The *HTT1a* transcript leads to production of the HTT1a protein, which initiates the aggregation process in Huntington's disease mouse models (Fienko et al., 2022; Landles et al., 2021). A similar dynamic, whereby mutant N-terminal fragments appear early and eventually form aggregates, is also observed in human Huntington's disease brains (Marcellin et al., 2012). In summary, the minipig model demonstrates that HTT1a is produced and, in the cortex, has started to form HTT1a aggregates.

The KI-85Q-HD minipig thus represents a genetically precise and translationally relevant large-animal model of Huntington's disease.

It may also allow the study of somatic CAG repeat instability, an emerging therapeutic target. Age-dependent repeat expansions are detectable across tissues, enabling evaluation of interventions that stabilize repeat length (Aldous et al., 2024; Choi et al., 2024; Mathews et al., 2024 preprint). Although expansion levels remain modest compared with humans, they contrast sharply with the complete stability observed in another large-animal model, the OVT73 transgenic sheep (Handley et al., 2024), or the transgenic minipig (Askeland et al., 2018; Bai et al., 2021). A major limitation is the extended time required for phenotypic manifestation, reflecting the slow disease progression and long lifespan of the species. Additionally, for gene-editing and silencing therapies, the targeted genomic region must be highly conserved between pig and human for the intervention to be testable. Sequence divergence may restrict the applicability of certain human-designed therapies or necessitate re-design of targeting reagents, representing a potential hurdle for translational studies. Despite these limitations, the KI-85Q-HD minipig in some ways bridges the translational gap between rodents and humans.

Looking ahead, the establishment of this KI-85Q-HD minipig opens new possibilities for therapeutic development. This model provides a powerful preclinical platform for testing genome-editing strategies designed to contract the CAG repeat expansion or selectively silence the mutant *HTT* allele (Lin et al., 2025), when the porcine targeting sequence is identical to the human gene. The KI-85Q-HD minipig offers a limited opportunity to investigate pharmacological or genetic interventions aimed at stabilizing the CAG repeat tract and limiting somatic expansion in peripheral and central tissues, due to the modest frequency and slow progression of somatic expansion in our model. In addition, therapeutic strategies targeting *HTT* splicing, such as those aimed at suppressing the production of *HTT1a* transcripts, can be evaluated by quantifying *HTT1a* mRNA and protein levels before and after treatment.

In summary, the KI-85Q-HD minipig exhibits key molecular hallmarks of Huntington's disease, such as full-length mutant HTT expression, somatic CAG repeat instability, production of HTT1a, and evidence of early-stage HTT aggregation (Fig. 6), although somatic expansion in the brain is less pronounced than in human Huntington's disease, likely reflecting species-specific differences in repeat instability mechanisms. This model could be a valuable translational tool for studying the biodistribution of novel biological therapeutics targeting HTT-lowering and genome-editing approaches.

## MATERIALS AND METHODS

### Huntington's disease KI minipig model generation and tissue collection

The KI-85Q-HD minipig model was generated at Exemplar Genetics (Iowa, USA) by genetically modifying dermal fibroblasts derived from Libechov minipigs. CRISPR/Cas9 genome editing was used to expand the endogenous *HTT* allele (CAG)14CAA(CAG)2 to (CAG)82CAA(CAG)2. Somatic cell nuclear transfer was used to generate F0 piglets. Two founder boars were transferred to Libechov (Czech Republic) to establish a breeding colony (F1-F3 generations). Age-matched WT and KI-85Q-HD littermates (*n*=3-4 per genotype at each age; Table S1) were perfused with ice-cold PBS. Brain and peripheral tissues were dissected, snap-frozen, and stored at −80°C. Additional F1 Libechov×Yucatan animals generated at Exemplar Genetics were used for analyses of repeat instability in liver and spleen at 9, 15 and 49 months. The full list of samples is provided in Table S1.

### Ethical approval

All animal procedures were approved by the Animal Care and Use Committee and the Ministry of Agriculture of the Czech Republic (protocol number 53/2015). All procedures were performed in accordance with national and institutional guidelines, and every effort was made to minimize animal suffering. The animals were housed in individual indoor cages with straw bedding and enrichment materials (e.g. balls, chains). They were provided with free access to water and received a standard diet twice daily. The housing conditions were in accordance with institutional guidelines.

### Genomic DNA isolation

Genomic DNA was extracted from frozen tissues using the NucleoSpin Tissue kit (Macherey-Nagel), DNeasy Blood and Tissue Kit (QIAGEN), and from whole blood and from FACS-sorted blood cells by phenol-chloroform extraction (Thermo Fisher Scientific), following manufacturers' protocols.

### PCR-based genotyping

*HTT* exon 1 was amplified using the genotyping primer set (Table S2) with Quick-Load Taq Master Mix (NEB). Genotyping primers target positions 1809912-1809936 and 1810121-1810147 of the NC_010450.4 reference sequence (Sscrofa11.1 Primary Assembly) and flank the CAG repeat-containing region in *HTT* Exon 1. Thermocycling conditions were as follows: initial denaturation at 95°C for 5 min; 35-40 cycles of 95°C for 30 s, 65°C for 30 s and 72°C for 2 min; followed by a final extension at 72°C for 5-10 min. PCR products were resolved on 2% agarose gels and visualized with GelRed staining (Invitrogen).

### White blood cell isolation and sorting

A white blood cell (WBC) suspension for sorting granulocyte, lymphocyte and monocyte populations was prepared by erythrocyte lysis of anticoagulated blood. Whole blood (4 ml) was lysed with 40 ml of 1× home-made lysis solution (150 mM NH_4_Cl, 10 mM NaHCO_3_, 1 mM EDTA disodium salt) for 10 min at room temperature (RT). The preserved WBCs were centrifuged at 700 ***g*** for 5 min, washed with PBS containing 1% fetal bovine serum (FBS), and diluted to twice the initial blood volume to generate a pre-sort suspension.

For immunolabeling of T-cell subsets, 4 ml of whole blood were stained with directly conjugated monoclonal antibodies (Table S3). Staining was performed at RT for 1 h in the dark. After staining, erythrocytes were lysed again with 40 ml of 1× lysis buffer for 10 min at RT, and labeled WBCs were pelleted (700 ***g***, 5 min) and resuspended in PBS+1% FBS to double the original blood volume. Samples were kept at RT for ≤30 min before sorting.

Cell sorting was performed on FACSAria™ SORP cell sorter (BD Biosciences). For unstained pre-sort samples, the acquisition rate was maintained below 10,000 events s^−1^ in PBS+1% FBS, and the forward scatter threshold was set to exclude erythrocyte debris and platelets. The gating hierarchy defined the overall cell population, which was subdivided into granulocyte, lymphocyte and monocyte populations. For antibody-stained samples, the forward scatter threshold and acquisition rate (<10,000 events s^−1^) were similarly optimized. The gating hierarchy included total CD3e^+^ T lymphocytes and specific gates for T helper lymphocytes (CD3^+^CD4^+^CD8^−^), cytotoxic T lymphocytes (CD3^+^CD4^−^CD8^+^), and double-positive T lymphocytes (CD3^+^CD4^+^CD8^+^) populations on a CD4 versus CD8 dot plot. Cells were sorted in Purity mode (≥85%) into 1.5 ml FBS-precoated tubes (to minimize adhesion). Approximately 300,000 cells were collected per population; a 60 μl aliquot (∼10%) was used to verify sort efficiency. Remaining cells were pelleted (700 ***g***, 5 min), and pellets were stored at −70°C for genomic DNA isolation.

### Semen collection and processing

Semen was collected manually, diluted with Beltsville thawing solution extender (Selko), centrifuged at 600 ***g*** for 20 min at 24°C, washed and centrifuged again for 10 min at 600 ***g***. The pellet was snap frozen at −80°C for further use.

### Analyses of CAG repeat expansion in bulk genomic DNA

The *HTT* CAG repeat was amplified using the genotyping primer set (Table S2) and Platinum™ Taq High Fidelity polymerase (Thermo Fisher Scientific). The forward primer was fluorescently labeled with 6-FAM. Thermocycling conditions were: 94°C for 2 min; 38 cycles of 94°C for 15 s, 58°C for 30 s and 68°C for 30 s; followed by a final extension at 68°C for 3 min. PCR products were resolved by capillary electrophoresis on an Applied Biosystems 3730xl DNA Analyzer using GS500LIZ internal size standard, and fragment lengths were analyzed using GeneMapper v5 software (Applied Biosystems). The length of the CAG repeats [‘n’ in the (CAG)nCAA(CAG)2 tract] was determined relative to a sequenced minipig standard with a (CAG)14CAA(CAG)2 repeat. A somatic expansion index was determined as previously described (Lee et al., 2010) relative to the modal allele of each sample and using a 1% relative peak height threshold cut-off. In some animals, the modal CAG length differed between tissues, typically by one CAG. We were unable to directly size the repeat in the tissues or cells relative to the repeat length of the animal at birth (i.e. inherited repeat length). As a result, we are unable to account for shifts, either up or down, in the modal repeat in any particular tissue or cell type relative to the inherited repeat and therefore may under- or over-estimate expansion as a consequence. This may mask our ability to detect very subtle differences in expansion between tissues/cell types.

### SP-PCR and KI-specific SP-PCR

SP-PCR was used to detect low frequency CAG expansions. Reactions were initially performed using 180 pg of genomic DNA corresponding to approximately 30 genome equivalents of genomic DNA (one diploid genome equivalent=6 pg genomic DNA), and template input was further empirically lowered as needed to achieve on average one amplifiable KI allele per PCR reaction. Reactions were repeated up to 80 times per sample, including the blanks. Expansion frequency was quantified only in positive reactions. Nested PCR was performed with SP-PCR primer set 1 (outer) and SP-PCR primer set 2 (inner) (Table S2), targeting the region flanking the pig *HTT* gene and amplifying both the WT and KI alleles. KI-specific SP-PCR was performed, using a forward primer targeting the LoxP-containing sequence of the KI allele to selectively amplify the mutant allele in the first (outer) PCR reaction [KI SP-PCR primer set 1 (outer)] and reverse primer SP-PCR primer set 2 (inner) (Table S2). Cycling conditions for the first PCR (SP-PCR primer set 1) were: 95°C for 5 min; 41 cycles of 95°C for 30 s, 58°C for 30 s and 72°C for 2 min; final extension at 72°C for 5 min. For the second PCR (SP-PCR primer set 2), conditions were identical except that the annealing temperature was raised to 63°C.

### Detection and quantification of HTT1a

RNA extraction and reverse transcription with oligo dT18 (Invitrogen) and PCR reactions were as previously described (Landles et al., 2024). Primers and probes (Table S4) and the predesigned TaqMan assays for minipig reference genes (*RPLP2*, *PPIA*, *UBC*, *CDKN1B*) were from Thermo Fisher Scientific. Plates were sealed with Microseal ‘B’ seals (Bio-Rad), centrifuged at 800 ***g*** for 30 s, and amplified in a Bio-Rad CFX Opus 384 Real time PCR machine as follows: 40 s at 95°C and 40× (7 s at 95°C, 20 s at 60°C).

### 3′RACE

Total RNA was extracted from frozen tissues using TRI Reagent (Sigma-Aldrich) and the RNeasy Mini Kit (QIAGEN), followed by DNase treatment. RNA integrity was verified by non-denaturing agarose gel electrophoresis and the Bioanalyzer RNA 6000 Pico Kit (Agilent). For cDNA synthesis, 1 μg total RNA was reverse-transcribed using the High-Capacity cDNA Reverse Transcription Kit (Applied Biosystems) with an oligo(dT)-adapter primer. To detect and quantify alternatively polyadenylated *HTT* transcripts, 3′RACE was performed using a *HTT* exon 1-specific forward primer and an adapter-specific reverse primer (3′RACE PCR primer set 1) to detect alternatively polyadenylated *HTT* transcripts generated by cryptic polyadenylation within intron 1. Nested PCR was carried out for enhanced specificity [3′RACE PCR primer set 2 (inner); all primers in Table S5]. PCR products of ∼600 bp were gel-purified and subjected to Sanger sequencing. Sequences were aligned to the *Sus scrofa HTT* locus using BLAST to confirm transcript identity and alternative polyadenylation sites.

### Antibodies

All antibodies used for western blotting and HTRF assays are listed in Tables S6, S7.

### SDS-PAGE and western blotting

Frozen tissues were homogenized and lysed in RIPA buffer containing protease and phosphatase inhibitors. Lysates were sonicated, centrifuged (20,000 ***g***, 15 min, 4°C), and supernatants (10 μg protein) were separated by SDS-PAGE (3-8% or 4-12% Tris-acetate gels) Proteins were transferred to nitrocellulose membranes, blocked in 5% milk and probed overnight at 4°C with anti-HTT (EPR5526, 1:2000) and anti-polyQ (3B5H10, 1:3000; or 5TF1-1C2, 1:2000) antibodies (Table S6). Total protein normalization was performed using MemCode staining (Life Technologies).

### HTRF

The detection of HTT protein isoforms by HTRF was performed as previously described (Landles et al., 2021, 2024), with minor modifications including 20% (w/v) lysate concentration and the use of CHDI Foundation-provided HTT1a neoepitope (1B12, 11G2) antibodies (instead of MW8). Protein extraction was standardized across all tissue types by weight-to-volume (w/v) ratios followed by a bead-beating grinder and lysis system (Lysing Matrix D with zirconium silicate beads, FastPrep-24 instrument; MP Biomedicals). Given that total protein yields and ‘housekeeping’ protein expression vary significantly across organ systems, HTRF signals were interpreted as relative indicators of HTT1a and mutant *HTT* presence in each tissue rather than as direct quantitative comparisons across organs. All antibodies used in this study are summarized in Table S7.

### Detection of HTT isoforms by HTRF assay

HTRF-based detection of HTT isoforms was performed as previously described (Landles et al., 2021, 2024), with minor modifications including 20% (w/v) lysate concentration and the use of CHDI HTT P90 (1B12, 11G2) antibodies instead of MW8. Antibodies used in these assays are summarized in Table S7.

### Statistical analysis

Data were screened for outliers using Grubb's test, and outliers were removed before between-group comparisons and datasets were tested for a normal Gaussian distribution (Shapiro–Wilk test). Gene expression values were normalized to the geometric mean of the reference genes, and relative expression was calculated using the 2−ΔCt2 method. Statistical analysis was performed using either unpaired, two-tailed Student's *t*-test or one-way ANOVA with Tukey's post-hoc test or Dunnett's multiple comparison test. Graphs were prepared using GraphPad Prism (v10). *P*<0.05 was considered statistically significant.

## Supplementary Material

10.1242/dmm.052803_sup1 Supplementary information
