# Supplementary material for: Molecular features of a Huntington's disease knock-in minipig
Source: Dis Model Mech. 2026 May 26;19(5):dmm052803. doi: 10.1242/dmm.052803 (PMC13267775; doi:10.1242/dmm.052803)
Supplement: Supplementary information [file dmm-19-052803-s1.pdf]

Table S1. Tissue Sampling of wild-type and Knock-In (85Q) Huntington's disease minipigs.

Age of minipigs	ID	Genotype	Sampled Tissues	Analysis	Number of CAGs	Sex
9 m	7191	WT	Striatum<br>Liver<br>Spleen	N/A	15/18<br>11/14<br>11/14	female
9 m	7192	WT	Striatum	N/A	15/18	N/A
9 m	7193	KI	Liver<br>Spleen	Instability	82/14<br>82/14	female
9 m	7195	KI	Liver<br>Spleen	Instability	81/14	male
9 m	7197	WT	Striatum	N/A	15/18	N/A
9 m	7190	KI	Striatum	Instability	15/82	male
9 m	7190	KI	Striatum<br>Liver<br>Spleen	Instability	15/81<br>83/14<br>83/14	male
15 m	A2625	WT	Striatum<br>Liver<br>Spleen	N/A	15/18<br>11/14<br>11/14	female
15 m	A4081	WT	Striatum	N/A	15/18	N/A
15 m	A4084	WT	Striatum	N/A	15/18	N/A
15 m	A2626	KI	Striatum<br>Liver<br>Spleen	Instability	15/82<br>83/14<br>83/14	female
15 m	A4083	KI	Striatum<br>Liver<br>Spleen	Instability	15/84<br>84/14<br>84/14	female
15 m	A4082	KI	Striatum<br>Liver<br>Spleen	Instability	FAILED<br>85/14<br>85/14	female
18 m	A14	KI	Sperm	Instability	14/83	male
27 m	A20		Blood	Instability	14/86	female
18 m<br>24 m<br>27 m	A21	KI	Sperm<br>Blood	Instability	14/86	male
18 m<br>54 m	A28	KI	Sperm	Instability	14/83<br>14/84	male
27 m	A34	KI	Blood	Instability	11/84	female
27 m	A35	KI	Blood	Instability	11/81	female
49 m	A4640	KI	Liver<br>Spleen	Instability	82/14<br>82/14	female
49 m	A4641	KI	Liver<br>Spleen	Instability	82/14<br>82/14	female
36 m	A142 (WT1)	WT	Putamen, Motor cortex, Somatosensory cortex, Insular cortex, Spinal cord, Mixed tissue.	HTRF<br>qPCR	18/18	male

Disease Models & Mechanisms • Supplementary information

			Caudate nucleus, Parietal cortex, Frontal cortex, Cerebellum, Bladder, Kidney, Nucleus accumbens, Liver, Skin, Spleen			
36 m	A143 (KI1)	KI	Putamen, Motor cortex, Somatosensory cortex, Insular cortex, Spinal cord, Mixed tissue. Caudate nucleus, Parietal cortex, Frontal cortex, Cerebellum, Bladder, Kidney, Nucleus accumbens, Liver, Skin, Spleen	Instability HTRF SP-PCR qPCR 3'RACE	18/85	male
36 m	A144 (KI2)	KI	Putamen, Motor cortex, Somatosensory cortex, Insular cortex, Spinal cord, Mixed tissue. Caudate nucleus, Parietal cortex, Frontal cortex, Cerebellum, Bladder, Kidney, Nucleus accumbens, Liver, Skin, Spleen	Instability HTRF SP-PCR qPCR 3'RACE	18/90	male
36 m	A146 (WT2)	WT	Putamen, Motor cortex, Somatosensory cortex, Insular cortex, Spinal cord, Mixed tissue. Caudate nucleus, Parietal cortex, Frontal cortex, Cerebellum, Bladder, Kidney, Nucleus accumbens, Liver, Skin, Spleen	HTRF qPCR	18/18	female
36 m	A147 (WT3)	WT	Putamen, Motor cortex, Somatosensory cortex, Insular cortex, Spinal cord, Mixed tissue. Caudate nucleus, Parietal cortex, Frontal cortex, Cerebellum, Bladder, Kidney, Nucleus accumbens, Liver, Skin, Spleen	HTRF qPCR	18/18	female
36 m	A 148 (KI3)	KI	Putamen, Motor cortex, Somatosensory cortex, Insular cortex, Spinal cord, Mixed tissue. Caudate nucleus, Parietal cortex, Frontal cortex, Cerebellum, Bladder, Kidney, Nucleus accumbens, Liver, Skin, Spleen	Instability HTRF SP-PCR qPCR 3'RACE	18/18	female
36 m	A149 (KI4)	KI	Putamen, Motor cortex, Somatosensory cortex, Insular cortex, Spinal cord, Mixed tissue. Caudate nucleus, Parietal cortex, Frontal cortex, Cerebellum, Bladder, Kidney, Nucleus accumbens, Liver, Skin, Spleen	Instability HTRF SP-PCR qPCR 3'RACE	18/85	male
36 m	A150 (WT4)	WT	Putamen, Motor cortex, Somatosensory cortex, Insular cortex, Spinal cord, Mixed tissue. Caudate nucleus, Parietal cortex, Frontal cortex, Cerebellum, Bladder, Kidney, Nucleus accumbens, Liver, Skin, Spleen	HTRF qPCR	18/87	male
36 m	A135 (KI6)	KI	Putamen, Motor cortex, Somatosensory cortex, Insular cortex, Spinal cord, Mixed tissue. Caudate nucleus, Parietal cortex, Frontal cortex, Cerebellum, Bladder, Kidney, Nucleus accumbens, Liver, Skin, Spleen	HTRF SP-PCR qPCR 3'RACE	18/85	male

51 m	H9657	–	Semen	Instability	11/83	male
51 m	H9658	–	Semen	Instability	11/82	male
81 m	H49658	KI	Putamen, Motor cortex, Somatosensory cortex, Insular cortex, Spinal cord, Mixed tissue. Caudate nucleus, Parietal cortex, Frontal cortex, Cerebellum, Bladder, Kidney, Nucleus accumbens, Liver, Skin, Spleen	SP-PCR	15/18	male

m = months, KI = knock-in, WT = wild-type, HTRF = homogeneous time resolved fluorescence, SP-PCR = small pool PCR, qPCR = real-time quantitative PCR, 3'RACE = 3' rapid amplification of cDNA ends.

Table S2. Summary of primers used for small-pool PCR and KI-specific small-pool PCR.

Primer set	Name	Annealing temperature	Sequence
**Genotyping primer set**	HTT-F	58°C and 65°C	5′ - ATG GCG ACC CTG GAA AAG CTG ATG A - 3′
	HTT-R	58°C	5′ - GGT CGG TGC AGG GGC TCC TCG GCC AC - 3′
**SP-PCR primer set 1 (outer)**	p HTT – F (5cds)	58°C	5′ - CGA CCC TGG AAA AGC TGA TG - 3′
	p HTT-R (685atg)	58°C and 65°C	5′ - AAC GGG AGC GCA GAC AAG - 3′
**SP-PCR primer set 2 (inner)**	p HTT-F (35atg)	63°C	5′ - AGT CTC TCA AGT CCT TCC AGC - 3′
	HTTSr6	63°C	5′ - AGGGGGCCCGCACTCAC - 3′
**KI SP-PCR primer set 1 (outer)**	KI-mHTT-F	58°C	5′ - ACA TTA TAC GAA GTT GTC GCC G - 3′
	HTTSr6	58°C	5′ - AGGGGGCCCGCACTCAC - 3′

SP-PCR = small pool PCR, KI = knock-in.

Table S3. Summary of antibodies used for blood cells immunolabeling.

Antibody	Name	Dilution	Catalog number
**anti-CD4 mAb**	PerCP-Cy™5.5 anti-pig CD4a	5 µL/100 µL	561474, BD Biosciences
**anti-CD3 mAb**	Alexa647 anti-pig CD3e	1 µL/100 µL	561476, BD Biosciences
**anti-CD8 mAb**	FITC Mouse anti-pig CD8a	1 µL/100 µL	551303, BD Biosciences

mAB = monoclonal antibody.

Table S4. Summary of primers used for qPCR.

Assay	Primer/probe	Sequence
**5' Int1.1**	Fw Primer	GTTGGTCTGCAGGCTCTT
	Probe	TGCGCTGTTTAGGTGGTGATGAGT
	Rv Primer	GACCCAATACTCCAGGCAAA
**5' Int1.3**	Fw Primer	GAGTCACGAGGGAGAAACTAAA
	Probe	ATTGGGTCGAGTTGACTGTGTGCA
	Rv Primer	TCAGCAACACGTCCTTCTC
**5' Int1.4**	Fw Primer	AAGGCGCTCACTCTGTTAAG
	Probe	ACGAAATCTCTGACACAGCAAGGATCC
	Rv Primer	AAATCACTCCTCGACTCTCATTT
**5' Int1.5**	Fw Primer	TGAGGACGGCTTTCAAGAAG
	Probe	ATCAGCCTTAACAGAGTGAGCGCC
	Rv Primer	GAAATCTCTGACACAGCAAGGA
**3' Int 1.1**	Fw Primer	CTTGGGAGGTTCTTCTTCCTT
	Probe	AGGTCTGAAGTGCCTTGTGCAGAG
	Rv Primer	GGCTCATAGAACTGTGCAAATC
**Ex 65 - 66.1**	Fw Primer	CCAGTTCCTGGATGACTTCTT
	Probe	TTTCTGTCCAACCAGCAGCCGTA
	Rv Primer	GTCTGGAACACCTGGTACAC

Table S5. Summary of Primers used in 3′ RACE.

Primer set	Name	Sequence
**Oligo(dT) (Integrated DNA Technologies)**	oligo(dT)-adapter primer	5′–GGC CAC GCG TCG ACT AGT ACT TTT TTT TTT TTT TV–3′
**3′ RACE -PCR primer set 1 (outer)**	Htt-F int438 (HTT exon 1)	5′–GTT GGT CTG CAG GCT CTT–3′
	adapter-specific-R1	5′–GGC CAC GCG TCG ACT AGT AC–3′
**3′ RACE -PCR primer set 2 (inner)**	Htt-F int782 (HTT exon 1)	5′–GTG GTT AAC GAA CCC GAC TAG GAA–3′
	adapter-specific-R2	5′–GGC CAC GCG TCG ACT AGT AC–3′

Table S6. Summary of antibodies used for western blotting.

Antibody	Target / Epitope	Dilution	Species / Clonality	Catalog number/ Source
**EPR5526**	Huntingtin	1:2000	Rabbit monoclonal	ab109115; Abcam
**3B5H10**	Mutant HTT (polyQ-expanded); polyglutamine stretch (Q66)	1:3000	Mouse monoclonal	MABN821; Sigma-Aldrich
**5TF1-1C2**	PolyQ-expanded proteins; Homopolymeric glutamine tract (≥37Q)	1:2000	Mouse monoclonal	MAB1574; Millipore

Disease Models & Mechanisms • Supplementary information

Table S7. Summary of Antibodies used in HTRF assays.

Name	Immunogen	Epitope	Species	Reference / Source
**2B7**	Human HTT peptide: aa 1-17(Paganetti et al., 2009)	LMKAFE*	Mouse monoclonal	CHDI Foundation
**MW1**	Human HTT Exon1 (67Q)(Ko et al., 2001)	PolyQ	Mouse monoclonal	CHDI Foundation
**MW8**	AEEPLHRPK (67Q)(Ko et al., 2001)	Within: AEEPLHRP4 Ends at proline	Mouse monoclonal	CHDI Foundation
**MAB5490**	Human HTT: aa 115-129(Lunkes et al., 2002)	Within: QSVRNSPEFQKLLGI (mouse L)	Mouse monoclonal	Sigma-Aldrich, MAB5490
**D7F7**	Synthetic peptide surrounding human HTT Pro 1220	Within: aa 1214-1223* QSDTSGPVTT (mouse A)	Rabbit monoclonal	Cell Signaling Technology #5656
**11G2**(Sapp et al., 2025)	C-terminal HTT exon 1 neoepitope peptide (AEEPLHRP-OH)	Within: AEEPLHRP-OH; terminates at proline	Rabbit monoclonal	CHDI Foundation
**1B12**(Sapp et al., 2025)	C-terminal HTT exon 1 neoepitope peptide (AEEPLHRP-OH)	Within: AEEPLHRP-OH; terminates at proline	Rabbit monoclonal	CHDI Foundation

*information provided by CHDI Foundation

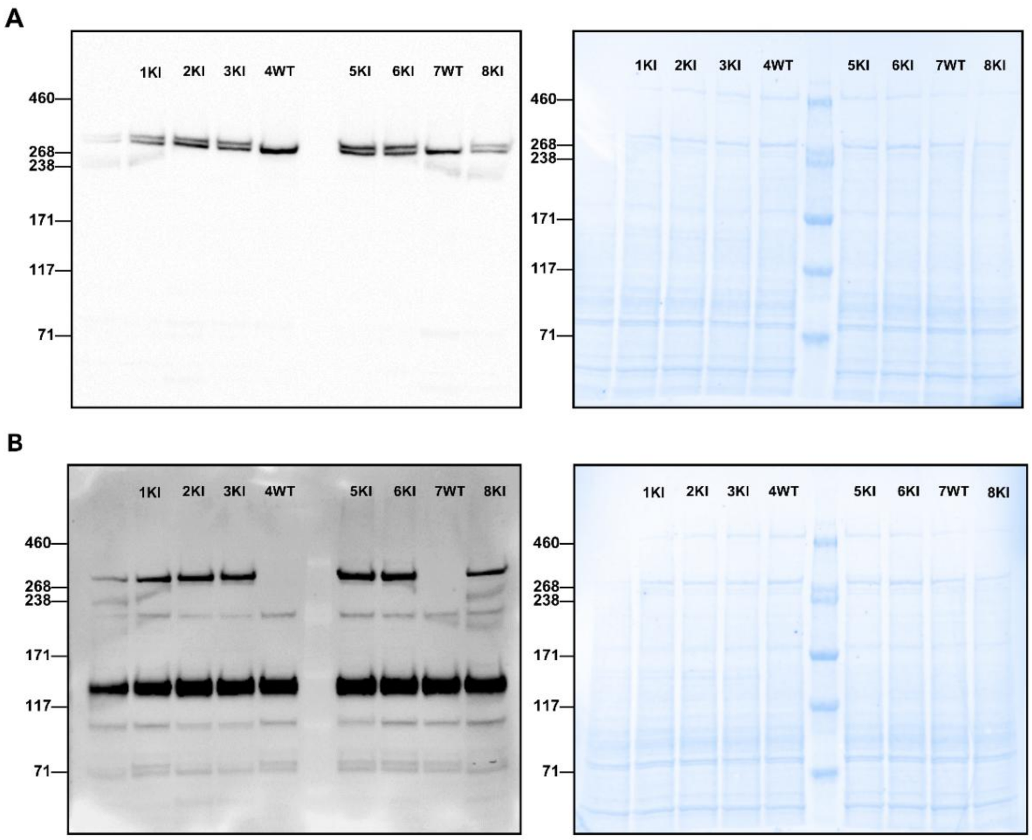

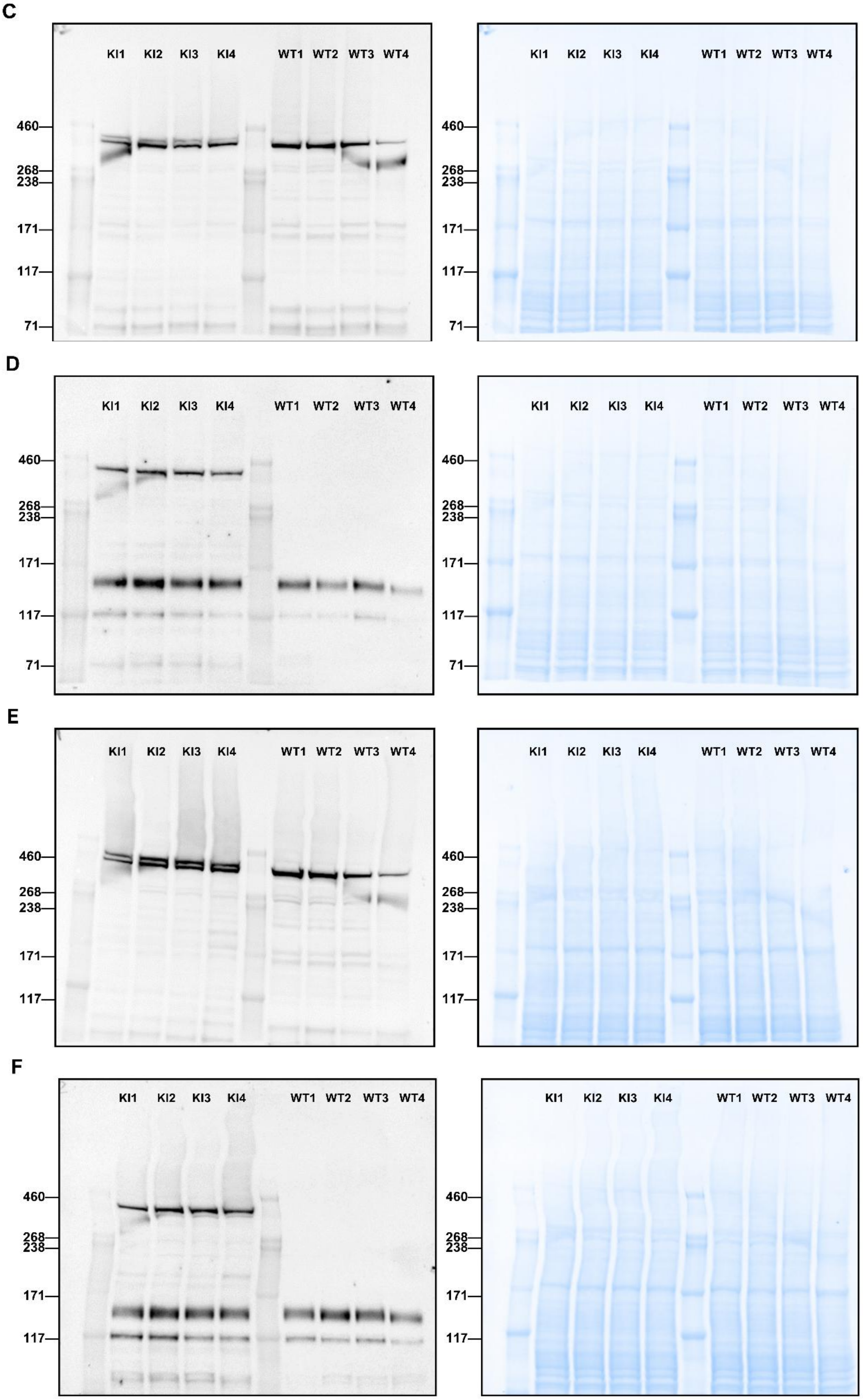

Fig. S1. Full-sized Western blot membranes and MemCode-stained loading controls corresponding to Fig.1C.

Full-length western blot membranes probed with antibody EPR5526 (left panels: A, C, E) and corresponding MemCode-stained membranes used as loading control (right panels: A, C, E)

from cortex tissue of minipigs at 6- (A), 12- (C), and 18- months of age (E). Full-length Western blot membranes probed with antibodies 1C2 or 3B5H10 (left panels: B, D, F) and corresponding MemCode-stained membranes used as loading control (right panels: B, D, F) from cortex tissue of minipigs at 6- (B), 12- (D), and 18- months of age (F). Size standards are in kDa. All antibodies used for western blotting are listed in **Supplementary Table 6.** KI = knock-in, mHTT = mutant HTT, WT = wild-type.

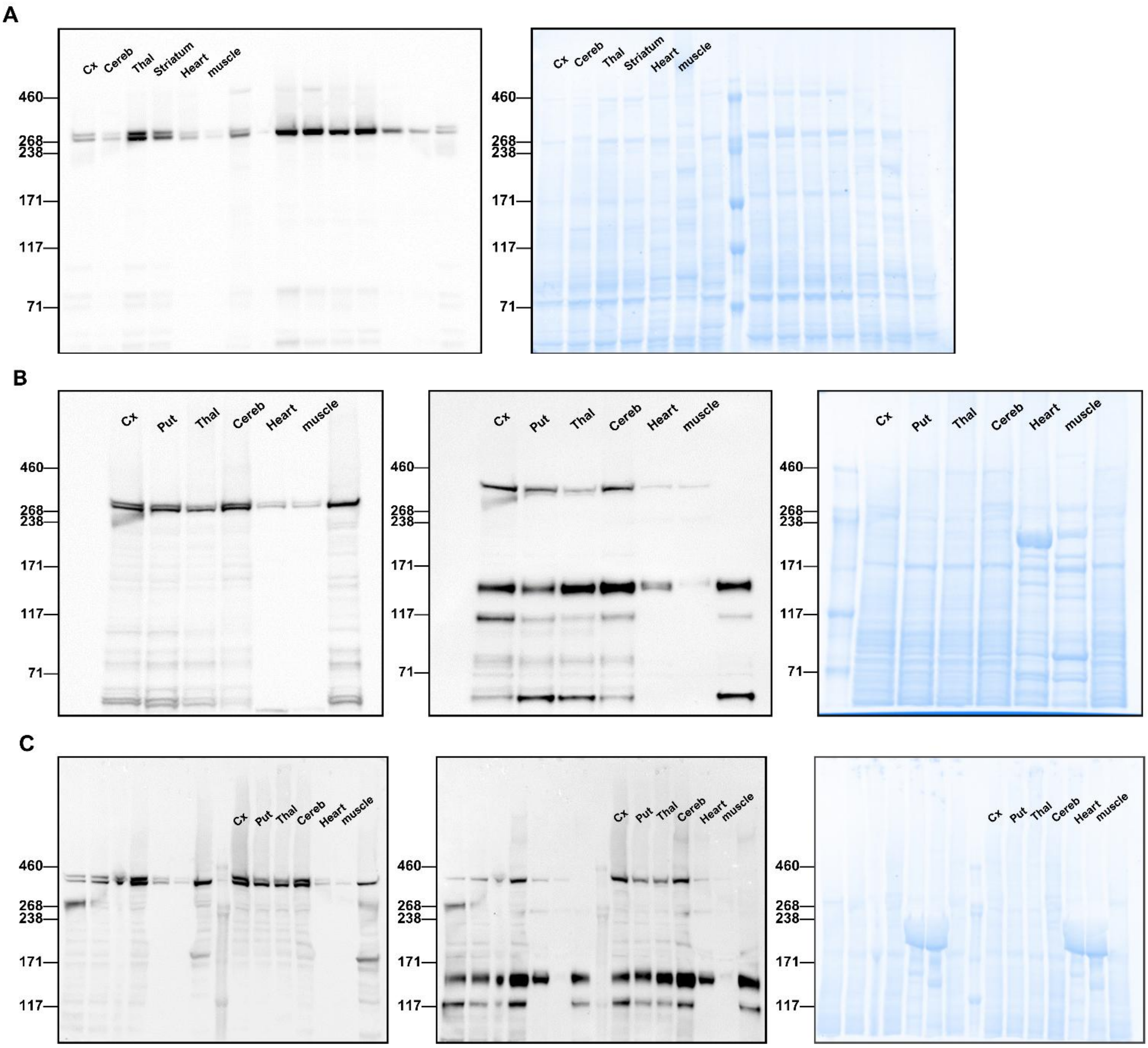

Fig. S2. Full-sized western blot membranes and MemCode-stained loading controls corresponding to Fig.1D.

(A) Full-length western blot membranes from 6-month-old minipigs. Left panel: probed with antibody EPR5526; right panel: corresponding MemCode-stained loading control. (B) Full-length western blot membranes from 12-month-old minipigs. Left panel: probed with antibody EPR5526; middle panel: probed with antibody 1C2 or 3B5H10; right panels: corresponding MemCode-stained loading control for both membranes. (C) Full-length western blot membranes from 18-month-old minipigs. Left panel: probed with antibody EPR5526; middle panels: probed with antibody 1C2 or 3B5H10; right panel: corresponding MemCode-stained loading control for both membranes. Size standards are in kDa. All antibodies used for western blotting are listed in Table S6. Cx = cortex, Put = putamen, Thal = thalamus, Cereb = cerebellum.

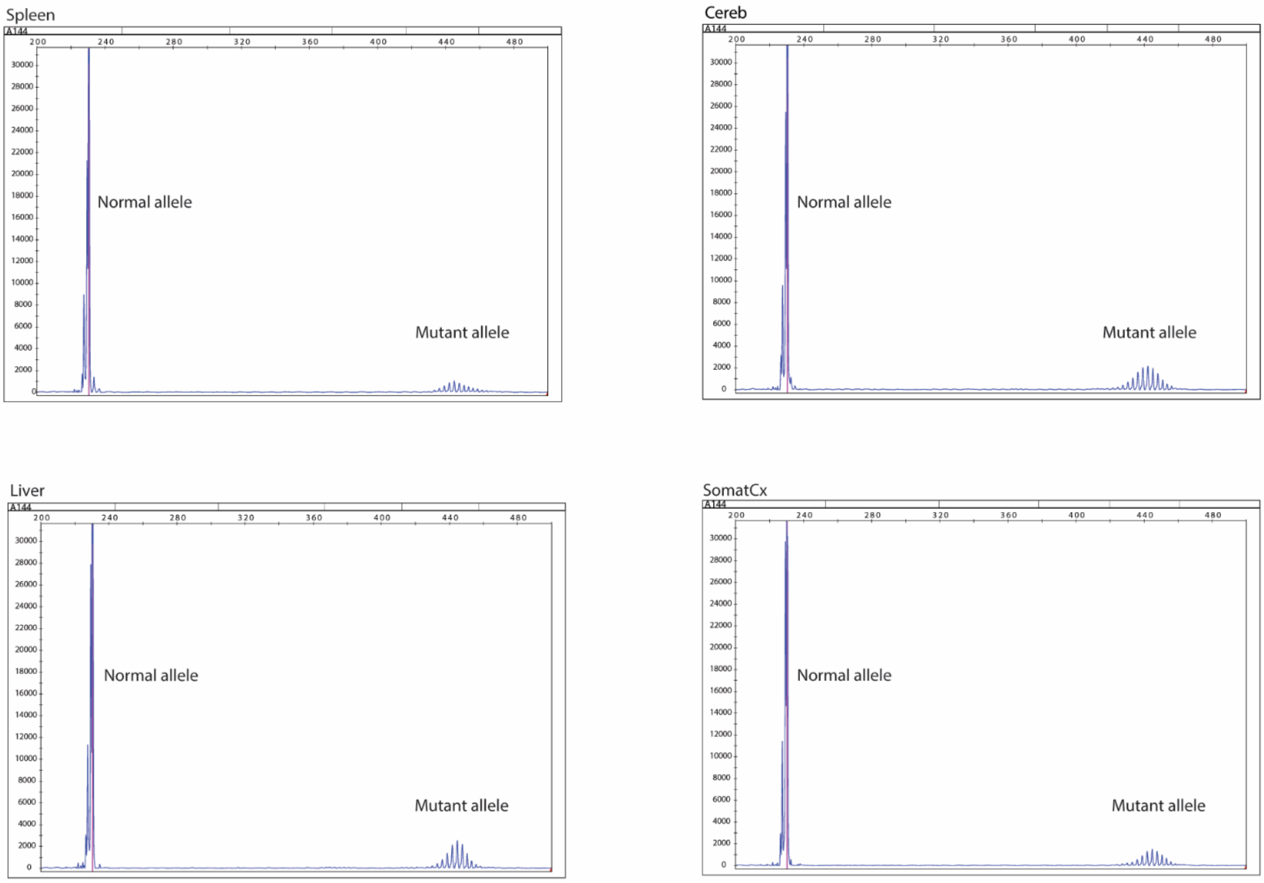

Fig. S3. *HTT* CAG PCR products from tissues of KI-85Q HD minipig KI2, highlighting somatic expansion of the mutant but not the wild-type allele.

Capillary electrophoresis traces from spleen, cerebellum (Cereb), liver, and somatosensory cortex (SomatCx) show two major *HTT* CAG PCR product signals corresponding to the wild-type allele and the mutant allele. The wild-type allele shows a single prominent peak, while the mutant allele displays a broadened, right-shifted distribution of multiple peaks. The expanded peak spread of the mutant allele reflects somatic CAG repeat instability, whereas the wild-type allele remains stable across tissues. These data illustrate somatic expansion of the minipig KI- 85Q knock-in allele.

References

Ko, J., Ou, S. and Patterson, P. H. (2001). New anti-huntingtin monoclonal antibodies: implications for huntingtin conformation and its binding proteins. *Brain Research Bulletin* **56**, 319–329.

Lunkes, A., Lindenberg, K. S., Ben-Haïem, L., Weber, C., Devys, D., Landwehrmeyer, G. B., Mandel, J.-L. and Trottier, Y. (2002). Proteases Acting on Mutant Huntingtin Generate Cleaved Products that Differentially Build Up Cytoplasmic and Nuclear Inclusions. *Molecular Cell* **10**, 259–269.

Paganetti, P., Weiss, A., Trapp, M., Hammerl, I., Bleckmann, D., Bodner, R. A., Coven-Easter, S., Housman, D. E. and Parker, C. N. (2009). Development of a Method for the High-Throughput Quantification of Cellular Proteins. *ChemBioChem* **10**, 1678–1688.

Sapp, E., Boudi, A., Iwanowicz, A., Belgrad, J., Miller, R., Robertson, R., O'Reilly, D., Yamada, K., Deng, Y., Joni, M., et al. (2025). Mutant Huntingtin exon1 protein detected in mouse brain with neoepitope antibody: effects of CAG repeat expansion, MSH3 silencing, and aggregation. *bioRxiv* 2024.12.31.630891.
